# Overcoming Low-Polarity Limitations in Polyphenylene Oxide Electrospinning: Chemical Functionalization and Polymer Hybridization for Interlaminar Toughening of Carbon Fiber Composites

**DOI:** 10.3390/polym17111480

**Published:** 2025-05-27

**Authors:** Yuan Huang, Yi Wei, Canyi Huang, Yiping Qiu, Bohong Gu, Bo Yang

**Affiliations:** 1 College of Textiles and Apparel, Quanzhou Normal University, Quanzhou 362000, China; yuanh@qztc.edu.cn (Y.H.); hcy126@qztc.edu.cn (C.H.); ypqiu@dhu.edu.cn (Y.Q.); gubh@dhu.edu.cn (B.G.); 2 State Key Laboratory of New Textile Materials and Advanced Processing Technology, School of Textile Science and Engineering, Wuhan Textile University, 1 Yangguang Road, Wuhan 430200, China; 3 Key Laboratory of Clothing Materials of Universities in Fujian, Quanzhou Normal University, Quanzhou 362000, China; 4 Center for Civil Aviation Composites, Donghua University, 2999 North Renmin Road, Shanghai 201620, China; weiy@dhu.edu.cn

**Keywords:** polyphenylene oxide, electrospun veils, sulfonation modification, physical blending, fracture toughness

## Abstract

This study investigates the optimization of polyphenylene oxide (PPO) electrospinning for interlaminar toughening in composites, using sulfonation modification and physical blending with polylactic acid (PLA) and polystyrene (PS). Both strategies showed excellent electrospinning performance, significantly reducing fiber diameter (PPO: 12.1 ± 5.8 μm; sulfonated PPO: 524 ± 42 nm; PPO-PLA: 4.73 ± 0.94 μm; PPO-PS: 3.43 ± 0.34 μm). In addition, the PPO-PS fibers were uniform, while PPO-PLA exhibited a mixture of fine and coarse fibers due to phase separation. Interlaminar fracture toughness testing showed that PPO-PS offered the greatest toughening, with *G_IC_^ini^* and *G_IC_^pre^* increasing by 223% and 232%, respectively, compared to the values of the untoughened sample, and by 65% and 61.5% compared to those of the PPO sample. *G_I__IC_* of the PPO-PS sample was 196% greater than that of the untoughened sample and 30% higher than that of the PPO sample. Scanning electron microscope (SEM) analysis of fracture morphology revealed that the high-toughness system dissipated energy through fiber bridging, plastic deformation, and multi-scale crack deflection, while the low-toughness samples failed due to interface debonding or cohesive failure. This work demonstrates that PPO-PS veils enhance interlaminar toughness through interface reinforcement and multiple toughening mechanisms, providing an effective approach for high-performance composites.

## 1. Introduction

Carbon fiber-reinforced polymers (CFRPs) occupy a significant position in fields such as aerospace and rail transportation due to their high specific strength, corrosion resistance, and design flexibility [1,2]. However, laminated structures are susceptible to delamination failure under impact or cyclic loading conditions, as well as due to manufacturing defects, which leads to a significant reduction in their damage tolerance. This presents a major technological challenge that limits their engineering implementation [3,4,5]. To enhance fracture toughness, multiple toughening strategies have been developed. Early-stage researchers incorporated rubber elastomers into epoxy matrices [6,7], inspired by polymer blending modification theory, achieving toughening through nanoscale phase separation that produced distinctive “island–sea” morphologies. While this strategy effectively improved composite fracture toughness, the introduction of rubber phases frequently induced concurrent modulus reduction and depression of glass transition temperatures (*T_g_*), rendering the modified systems inadequate to meet rigorous thermomechanical requirements in structural applications. The advent of thermoplastic toughening systems in the 1980s marked a paradigm shift, where high-performance thermoplastics typified by polyetherimide (PEI) and polysulfone (PSF) progressively supplanted rubber elastomers [8,9,10]. These materials achieve the simultaneous enhancement of interlaminar fracture toughness and preservation of composite rigidity/thermal stability through precisely controlled phase separation during curing, which generates either interpenetrating networks or phase gradient architectures within the matrix. However, the inherent viscoelastic disparity between thermoplastic constituents and thermosetting matrices induces an exponential escalation in the blend system melt viscosity, ultimately leading to significantly narrowed processing windows and compromised manufacturability in composite fabrication.

The advent of the 21st century has witnessed the emergence of innovative interlayer toughening strategies in academic research [11,12], encompassing the incorporation of thermoplastic particles [13], films [14], and electrospun fibers [15,16]. Recent advancements in nanomodified interlayers highlight both their functional potential and technical challenges. For instance, Mahato et al. [17] utilized carbon nanotube (CNT) masterbatch-coated glass fiber prepregs to develop conductive interleaves, reporting a 27% increase in mode I fracture toughness (DCB tests) at 7.5 wt% CNT loading, alongside real-time damage diagnosis via electrical resistance monitoring. Notably, this approach exhibited strong concentration dependence, as a 0.6 wt% CNT content reduced toughness by 80%, emphasizing the delicate balance required in particulate-based systems. In contrast, electrospun nanofibers inherently circumvent such dispersion-sensitive limitations through their continuous three-dimensional porous architectures (50–500 nm diameter), which mechanically reinforce interlaminar regions via crack deflection and stress redistribution, without relying on critical filler thresholds. Sumit et al. [18] exemplified this structural advantage by engineering electrospun thermoplastic scaffolds with dual-bonding interfaces, achieving 60% shear strength and 100% toughness improvements—a result attributed to covalent/non-covalent synergy rather than particulate loading optimization. Huang’s poly(arylene ether nitrile)-poly(epsilon-caprolactone) (PAEN-PCL) hybrid membranes [19] overcame toughness–rigidity trade-offs (132.5% mode I fracture toughness, 17.9% modulus) but emphasized electrospinning’s polarity dependency. Yu’s spray-coated carboxylated aramid nanofibers (cANFs) [20] bypassed the electrospinning limitations (24–74% interlaminar improvements) but sacrificed fiber alignment efficiency. These studies collectively reveal that (i) chemical functionalization enhances interfacial compatibility; (ii) hybridization enables multimodal toughening.

Polyphenylene oxide (PPO), a high-performance engineering thermoplastic that was developed in the 1960s, exhibits outstanding dimensional stability, low shrinkage/water absorption, and high-temperature creep resistance [21,22]. Compared to conventional toughening thermoplastics (e.g., polyamide (PA), polycaprolactone (PCL)), PPO offers unique advantages such as maintained mechanical stability above 200 °C and dimensional precision in humid environments with less than 0.1% moisture absorption. However, due to its low-polarity chemical structure, PPO exhibits poor solubility in polar solvents, which imposes limitations on fiber fabrication via electrospinning. Charge imbalance and fiber inhomogeneity are common issues during electrospinning [23]. Previous studies by the authors demonstrated that incorporating functionalized carbon nanotubes (FCNTs) could regulate PPO fiber diameter and enhance their electrospinnability [24]. FCNTs effectively reduced fiber diameter and improved the mode I and mode II fracture toughness of laminates. However, excessive FCNT content induced the formation of bead-like structures on the fiber surface, which compromised the toughening efficacy. This issue highlighted the limitations of relying solely on physical additive modification strategies. Therefore, it is imperative to systematically optimize the spinnability and toughening efficiency of PPO through chemical modification or physical blending methods in future work.

To address these challenges, this study proposes two modification strategies: first, incorporating polar sulfonic acid groups into the PPO backbone through sulfonation (yielding sulfonated PPO, SPPO) to enhance PPO solubility and charge balance capability during electrospinning; and second, blending PPO with highly polar, electrospinnable polymers (e.g., polylactic acid (PLA) and polystyrene (PS)), improving fiber formation uniformity by leveraging polarity gradients. PLA and PS were chosen for their solubility in chloroform—PPO’s optimal solvent—and their synergistic ability to address PPO’s low-polarity limitations. While alternatives such as PCL, polyethylene oxide (PEO), polyvinyl alcohol (PVA), and polyvinylidene fluoride (PVDF) were considered, their incompatibility with chloroform (e.g., PEO/PVA’s hydrophilicity, PVDF’s crystallinity) or thermal limitations (e.g., PCL’s low *T_g_*) rendered them unsuitable for this system. A comparative analysis of the morphological characteristics of pure PPO veils, SPPO veils, PPO/PLA veils, and PPO/PS veils revealed the influence of chemical modification and physical blending on fiber diameter and uniformity. The distinct toughening contributions of these modification strategies were further elucidated through mode I (double cantilever beam, DCB) and mode II (end-notched flexure, ENF) interlaminar fracture toughness tests. The experimental results revealed that the PPO/PS blend fibers exhibited the most pronounced toughening effect, with the observed improvement in fracture toughness being ascribed to a synergistic reinforcement at the fiber–matrix interface. This finding provides a new technological pathway for the development of high-performance interlayer toughening materials.

## 2. Experimental Section

### 2.1. Materials

The UIM17500 carbon fiber unidirectional prepreg (areal weight: 200 g/m^2^, resin content: 30–33%) was provided by Weihai Guangwei Composite Materials Co., Ltd. (Weihai, China). PPO 630 was supplied by SABIC Innovative Plastics (China) Co., Ltd. (Guangzhou, China). (particle size: 10–200 μm, Mn: 17,300, *T_g_*: 214 °C). PS was purchased from Shanghai Titan Technology Co., Ltd. (Shanghai, China). PLA was purchased from Wendong (Shanghai) Chemical Co., Ltd. (Shanghai, China). Chlorosulfonic acid (99%) and chloroform were purchased from Sinopharm Chemical Reagent Co., Ltd. (Shanghai, China). All materials were used without further purification.

### 2.2. Preparation of SPPO

The sulfonation degree of PPO is defined as the ratio of sulfonic acid groups (-SO_3_H) substituted on PPO molecules to the total number of PPO molecules. By varying reaction conditions and durations, PPO with distinct sulfonation degrees can be synthesized. A higher sulfonation degree indicates greater incorporation of sulfonic acid groups into the PPO molecular structure, thereby altering its physical and chemical properties. Low-sulfonation-degree PPO retains partial non-sulfonated benzene ring structures, preserving a favorable mechanical performance. To balance the retention of PPO’s mechanical properties with enhanced polarity, this study synthesized sulfonated PPO (SPPO) with controlled low sulfonation degrees.

**Figure 1:**
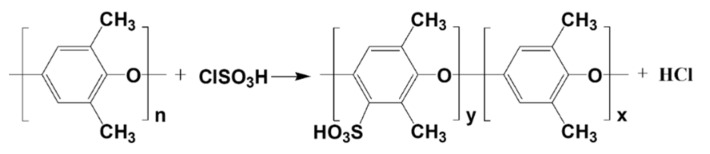
Reaction for the preparation of SPPO.

Following the methodology reported by Smitha et al. [25], sulfonated PPO was synthesized via a sulfonation reaction between PPO and chlorosulfonic acid in a chloroform solvent system. First, 50 mL of chloroform was added to a three-neck flask, followed by dissolving 5 g of PPO under continuous stirring at room temperature for 30 min to form a homogeneous solution. Subsequently, 5 mL of chlorosulfonic acid was mixed with 100 mL of chloroform, transferred to a separatory funnel, and gradually added dropwise into the PPO-containing flask over 20 min. A dark brown polymeric precipitate formed during the reaction. The product was washed with deionized water, dried in a vacuum oven at 50 °C for 4 h, and further treated in a conventional oven at 110 °C for 4 h. The reaction for SPPO preparation is illustrated in Figure 1.

### 2.3. Preparation of Modified-PPO Electrospun Veils

The electrospinning of the PPO, SPPO, PPO-PLA, and PPO-PS veils was performed using a standard single-nozzle electrospinning apparatus. Table 1 presents the compositions of the electrospinning solutions and the corresponding electrospinning parameters for each veil. The variations in solution concentration and processing parameters across materials primarily arose from differences in polymer solubility, viscosity, and chain entanglement behavior. These parameters were further refined through iterative experimental trials to identify their optimal combination ensuring stable fiber formation and minimal defects. According to the ratios provided in the table, the solutes were thoroughly dissolved in chloroform under magnetic stirring. The electrospinning setup was placed in a closed-environment box containing a silica gel desiccant, maintaining a relative humidity of approximately 50% and a temperature of 26 °C. The solution was supplied to the electrospinning apparatus through a stainless-steel needle (20 G, with inner and outer diameters of 0.58/0.88 mm) at a constant feed rate. To ensure consistency in the interlaminar toughening effect, all veils were designed with a target areal density of 5 ± 0.5 g/m^2^, as established in our prior study [26] to balance mechanical performance and material economy.

### 2.4. Preparation of Composite Laminates

For the DCB and ENF tests, unidirectional prepreg tapes were laid up in a [0°]_20_ ply configuration to fabricate composite laminates with dimensions of 250 × 220 mm^2^. During the lay-up process, the PPO, SPPO, PPO-PLA, and PPO-PS veils were placed between the 10th and the 11th plies of the prepreg, separately. A 13 μm thick PTFE film was placed in the middle layer at an appropriate position to introduce an initial crack, as per the testing standards. The prepreg lay-up was vacuum-bag-molded under the manufacturer’s recommended conditions and cured at 120 °C for 90 min. The four toughened samples were labeled as PPOv, SPPOv, PPO-PLAv, and PPO-PSv.

### 2.5. Testing and Characterization

#### 2.5.1. Characterization of SPPO

Surface functional group characterization of PPO and SPPO was conducted using a Nicolet 8700 Fourier transform infrared (FTIR) spectrometer from Thermo Fisher Scientific (Waltham, MA USA), in a wavenumber range of 4000–500 cm^−1^.

The measurements were conducted using a NETZSCH 214 differential scanning calorimeter (DSC) from TA Instruments (New Castle, DE, USA), under a N_2_ atmosphere.

Thermogravimetric analysis (TGA) of PPO and SPPO was performed using a DISCOVERY TGA550 analyzer from Thermo Fisher Scientific (Waltham, MA, USA). The samples were heated in a N_2_ atmosphere at a heating rate of 10 °C/min, from room temperature to 600 °C.

#### 2.5.2. SEM Characterization

The morphology of different veils was observed using a scanning electron microscope (SEM, Neo Scope JCM-6000Plus, JEOL, Tokyo, Japan). Prior to observation, the veils were gold-coated. The fiber diameter was measured using software, and the average diameter and standard deviation of the fibers were calculated using Gaussian fitting.

For fracture surface characterization, the microscopic fracture surfaces of the laminate reinforced with different veils were specifically examined using the same SEM system. Prior to SEM observation, the fracture surfaces of the laminates were gold-coated.

#### 2.5.3. Fracture Toughness Evaluation

Fracture toughness testing of the laminates was conducted on a 2 kN electromechanical universal testing machine (Wance ETM104B-EX, Beijing, China). DCB and ENF specimens were mounted on the machine and examined at a crosshead displacement rate of 1 mm/min. Load–displacement data were recorded during testing. Five replicates per sample group were tested, and the averaged values were used for analysis.

In accordance with ASTM D5528, one side of the DCB specimens was coated with white paint, and 1 mm interval marks were made from the PTFE film insertion point to *a*_0_ = 50 mm. Each specimen was unloaded after the first delamination growth increment. The load–displacement curve at this stage was recorded. Mode I crack initiation energy (*G_IC_^ini^*) was calculated using the 5% offset/maximum load method. This method involves drawing a line from the origin with a 5% compliance offset from the initial linear region of the load–displacement curve. If intersection occurs beyond the maximum load point, the maximum load is used; if it occurs prior to the maximum load, the intersection value is adopted. Without removing it from the grips, the specimen was reloaded until the final delamination length was achieved. Load, displacement, and crack length were recorded at each marked position. These data were used to calculate mode I propagation energy (*G_IC_^prop^*) at each point. The second loading cycle induced natural mode I precracks in the specimen, with the first *G_IC_^prop^* defined as mode I precrack energy (*G_IC_^pre^*). According to modified beam theory, mode I interlaminar fracture toughness *G_IC_* is determined by Equation (1):

(1) $$G_{IC}=\frac{3P_{c}\delta }{2b(a+\left|∆\right|)}\;$$

where P_c_ is the critical load at crack propagation (N), *δ* is the load-point displacement corresponding to P_c_ (mm), b is the specimen width (mm), a is the crack length (mm), and Δ is the crack length correction factor (mm). Δ was determined experimentally by plotting the cube root of compliance C^1/3^ against crack length and performing a least-squares fitting. The specimen dimensions were *b* = 20 mm, untoughened specimen thickness *h* = 3.14 mm, with a maximum thickness increase ≤8% after PPO veil insertion. Initial crack length *a*_0_ = 50 mm, total span L = 150 mm.

For ENF testing, load–displacement curves were recorded during experimentation. A load drop was observed upon crack propagation initiation. The first peak load and corresponding displacement were recorded to calculate mode II interlaminar fracture toughness *G_IIC_*. Data processing followed the methodologies described by Russell and Street [27], with the critical strain energy release rate calculated via Equation (2):

(2) $$G_{\mathrm{IIC}}=\frac{9P_{c}\delta a^{2}}{2b(2L^{3}+3a^{3})}\;$$

where Pc is the first peak load (N), *δ* is the load-point displacement at *P_c_* (mm), L is half-span length (50 mm), *b* is the specimen width (mm), and *a* is crack length (mm). The specimen dimensions were *b* = 20 mm, untoughened specimen thickness *h* = 3.12 mm; initial crack length *a*_0_ = 30 mm.

## 3. Results and Discussion

### 3.1. Characterization Results of SPPO

**Figure 2:**
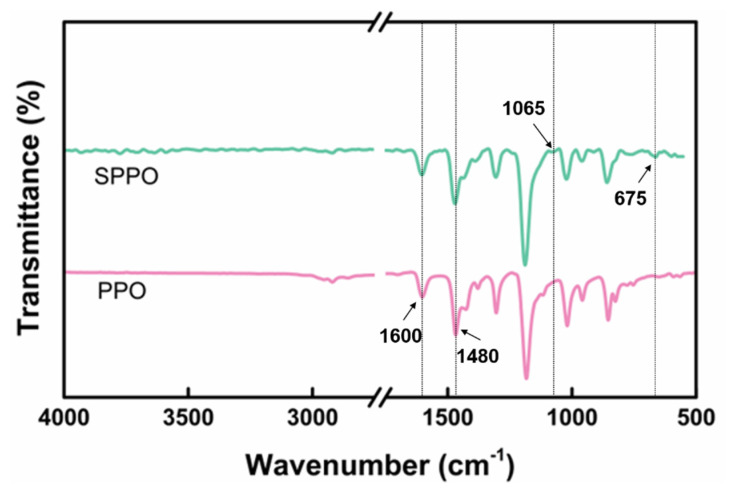
FTIR spectra of PPO and SPPO.

Figure 2 shows the FTIR spectra of PPO and SPPO. The spectra of all samples exhibited aromatic group bands around 1600 and 1480 cm^−1^, characteristic of PPO. In the SPPO spectrum, new absorption peaks at 675 cm^−1^ and 1065 cm^−1^ were observed, which correspond to the stretching vibrations of the -SO_3_ group and the S=O bond, respectively [28].

**Figure 3:**
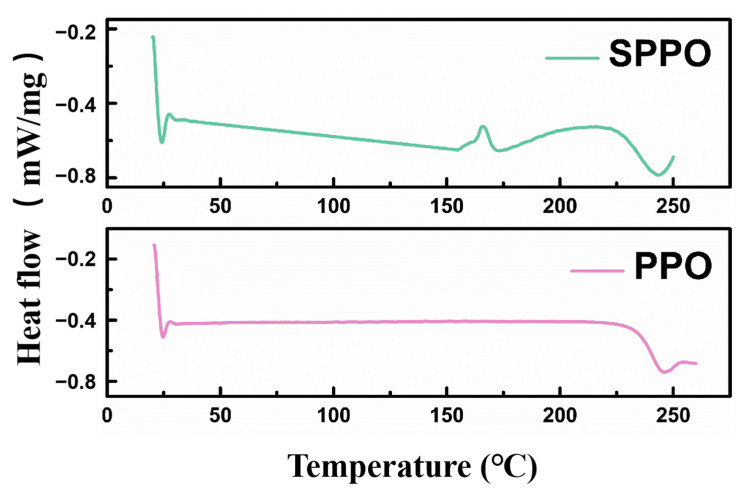
DSC of PPO and SPPO.

Figure 3 presents the DSC curves of the PPO and SPPO samples. The measurements were performed over a temperature range of 30–250 °C at a heating rate of 10 °C/min, using hermetically sealed aluminum pans. As a semi-crystalline polymer, PPO exhibits characteristic DSC thermograms revealing crystalline-phase melting behavior [29]. The unmodified PPO sample showed a distinct endothermic melting peak at 246 °C. The close proximity between the melting temperature (Tm) of PPO’s crystline phase and its Tg prevented a clear resolution of the Tg transition. The SPPO samples displayed nearly identical melting temperature ranges and peak positions to those of pure PPO. Notably, an exothermic peak emerged at 170 °C in the SPPO thermogram, which may correlate with the degradation or decomposition of sulfonic acid groups, as evidenced by corresponding transitions in the TGA profiles.

**Figure 4:**
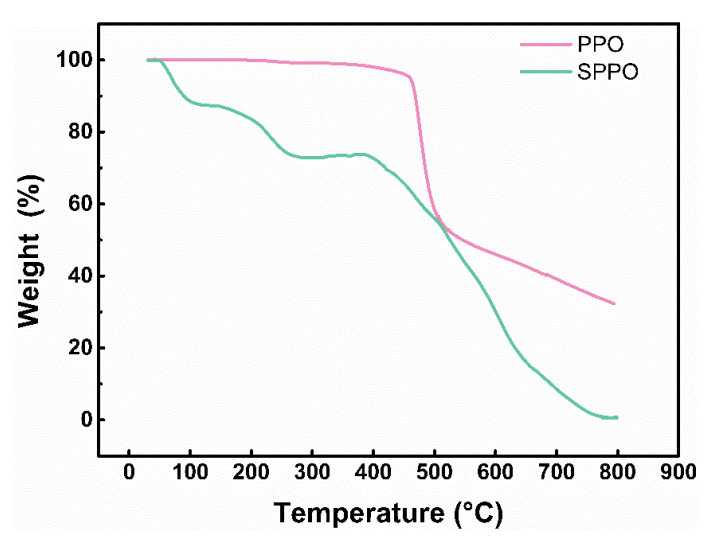
TGA of PPO and SPPO.

The thermal stability of pure PPO and SPPO was investigated by TGA, with the corresponding thermograms displayed in Figure 4. Prior to testing, all samples were dried at ambient temperature for several days. As observed in Figure 4, unmodified PPO exhibited a two-stage decomposition process. The first decomposition stage initiated at 450 °C with 46% mass loss and was followed by a second stage commencing at 500 °C. Beyond this temperature, the mass loss rate decreased progressively, resulting in 21% total mass loss and 33% residual char at 800 °C. The predominant mass loss occurring between 450 and 500 °C is likely attributable to backbone scission of the polymer chains, consistent with the thermal degradation temperature range reported for PPO [30]. In contrast, SPPO demonstrated three distinct mass loss stages within the temperature ranges of 50–120 °C, 170–280 °C, and 380–800 °C, with corresponding mass loss rates of 12%, 15%, and 72%, respectively. The initial polymer decomposition occurred at a temperature close to 400 °C. The temperature intervals of these degradation stages showed close agreement with those reported by Smitha et al. [25] and Petreanu et al. [31]. For the SPPO samples exposed to atmospheric conditions before TGA testing, the first mass loss stage (50–120 °C) corresponded to the evaporation of adsorbed moisture, the second stage (170–280 °C) arose from the decomposition of sulfonic acid groups, and the third stage (380–800 °C) resulted from backbone cleavage prior to complete polymer degradation. Notably, SPPO exhibited near-zero residual mass at 780 °C, indicating that sulfonation significantly deteriorated the thermal stability of PPO.

FTIR spectra analysis confirmed the successful incorporation of -SO3H into PPO through sulfonation modification. DSC characterization revealed that the material’s melting behavior and phase transition characteristics remained essentially unaltered. TGA demonstrated a marked decrease in the thermal stability of SPPO compared to that of pure PPO, which may adversely influence the curing kinetics of epoxy resin systems.

### 3.2. Morphology Characterization of the Electrospun Veils

**Figure 5:**
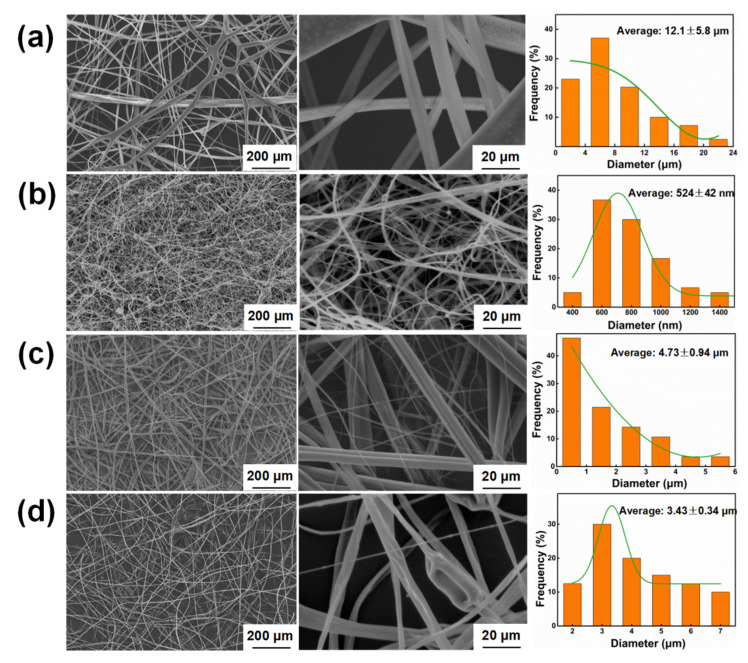
SEM micrograph and diameter distribution of four electrospun veils: (**a**) PPO; (**b**) SPPO; (**c**) PPO-PLA; (**d**) PPO-PS.

Figure 5 presents representative SEM micrographs of electrospun veils. Notably, the SPPO and blended PPO-PLA/PPO-PS veils demonstrated a substantial reduction in fiber diameter relative to the pure PPO veils (Figure 5a), indicating enhanced spinnability through both chemical modification and physical blending. A quantitative analysis revealed that the SPPO veils exhibited the finest fiber morphology, with an average diameter of 524 ± 42 nm and predominantly ribbon-like cross sections (Figure 5b). This phenomenon may have arisen from the polar sulfonic acid groups in SPPO, which improved solution homogeneity and stabilized the electrospinning jet, thereby promoting uniform fiber stretching. The blended systems showed intermediate diameter distributions: the PPO-PLA and PPO-PS veils displayed average fiber diameters of 4.73 ± 0.94 μm and 3.43 ± 0.34 μm, respectively. The PPO-PLA veils (Figure 5c) displayed a heterogeneous diameter distribution, with distinct populations of thick and thin fibers. This morphological complexity stemmed from the thermodynamic incompatibility between amorphous PPO and semi-crystalline PLA, which induced polymer-rich domain formation during electrospinning. A statistical analysis confirmed that the thin fibers predominantly originated from the PLA phases with enhanced chain mobility, while the thick fibers corresponded to the PPO phases. Notably, these thick PPO fibers in the blend had diameters that aligned with the main diameter range of pure PPO (5–15 μm), but the extreme outliers (>20 μm) observed for pure PPO (Figure 5a) were eliminated; this was likely due to PLA-enhanced jet stretchability, which suppressed extreme fiber coarsening by alleviating the localized elongational stress during electrospinning, while retaining PPO’s intrinsic propensity for aggregation-induced thicker fiber formation. In marked contrast, the PPO-PS veils (Figure 5d) exhibited monodisperse fiber distributions with smooth surfaces, reflecting an excellent miscibility between the amorphous PPO and PS phases. This compatibility suppressed phase separation, enabling uniform fiber formation. Although PPO-PS achieved effective fiber diameter refinement compared to pure PPO, sporadic bead defects were detected, suggesting partial instability in the electrospinning jet.

### 3.3. Results of Fracture Toughness Testing

#### 3.3.1. Mode I Interlaminar Fracture Toughness

**Figure 6:**
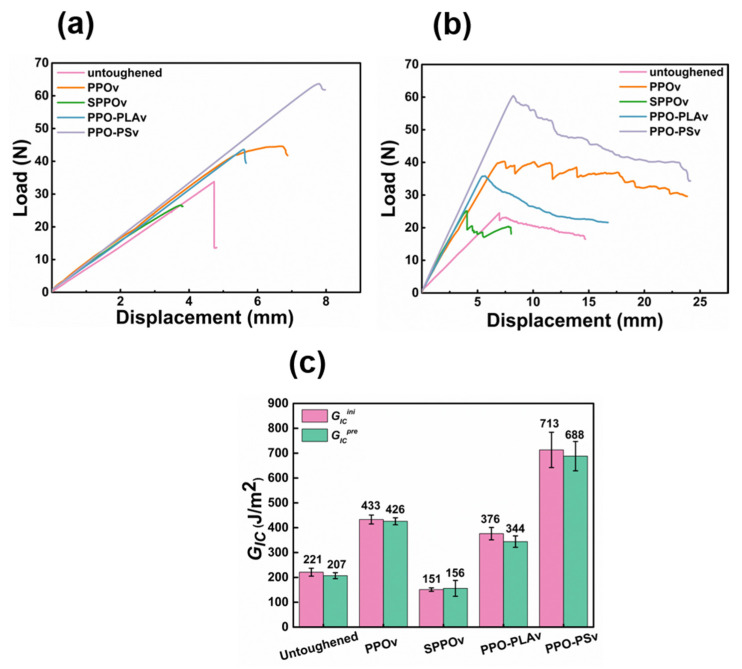
Results of DCB test on toughened laminates of four different electrospun veils: (**a**) load–displacement curves of first loading; (**b**) load–displacement curves of second loading; (**c**) values of *G_IC_^ini^* and *G_IC_^pre^*.

Figure 6 presents the mode I interlaminar fracture toughness test results for the untoughened laminate and four kinds of toughened laminates with different veil reinforcements. Figure 6a shows the load–displacement curve for the first loading cycle. The critical load and corresponding displacement at the loading point for the PPO-PSv sample were at a high level, indicating a further enhancement compared to the PPO veil, which already exhibited excellent toughening effects. Another noteworthy observation is that the critical load and corresponding displacement at the loading point for the SPPOv sample were significantly lower than those for the untoughened sample. The critical load and corresponding displacement for the PPO-PLAv sample were higher than those for the untoughened sample, but slightly lower compared to those for the PPOv sample. Figure 6b shows the load–displacement curve for the second loading cycle. Consistent with the results in Figure 6a, the load–displacement curve of the PPO-PSv sample remained at a relatively high level, indicating that crack propagation in the sample encountered higher resistance.

The mode I fracture toughness values of the samples are listed in Figure 6c, showing that the *G_IC_^ini^* and *G_IC_^pre^* values were quite similar, with minimal differences. The *G_IC_^ini^* and *G_IC_^pre^* values of the PPO-PSv sample reached 713 J/m^2^ and 688 J/m^2^, respectively, representing a 223% and 232% improvement compared to the untoughened sample’s values of 221 J/m^2^ and 207 J/m^2^. Compared to the PPOv sample, there was a further improvement of 65% and 61.5%, respectively. The SPPOv sample exhibited very poor interlaminar toughness, with *G_IC_^ini^* and *G_IC_^pre^* values of only 151 J/m^2^ and 156 J/m^2^. Previous studies have indicated that finer PPO fibers generally have enhanced toughening effects. However, the SPPOv sample, which had the smallest fiber diameter, demonstrated a reduction in toughness, suggesting that SPPOv may have caused defects in the laminate. The *G_IC_^ini^* and *G_IC_^pre^* values of the PPO-PLAv sample were 376 J/m^2^ and 344 J/m^2^, showing an improvement in fracture toughness compared to the untoughened sample, but a decrease relative to the PPOv sample. This discrepancy arose from two compounding factors. First, blending PLA with PPO decreased the effective volume fraction of PPO—the primary toughening component. Second, despite refining the fiber morphology (e.g., reduced average diameter), PLA’s inherent brittleness [32] and the phase-separated structure (Figure 5c) isolated PLA and PPO into discrete domains, thereby hindering synergistic toughening. Thus, the anticipated benefits of fiber refinement were offset by the combined effects of PPO dilution and PLA’s mechanical limitations, underscoring the challenge of concurrently optimizing processability and toughening efficacy in hybrid systems.

#### 3.3.2. Mode II Interlaminar Fracture Toughness

**Figure 7:**
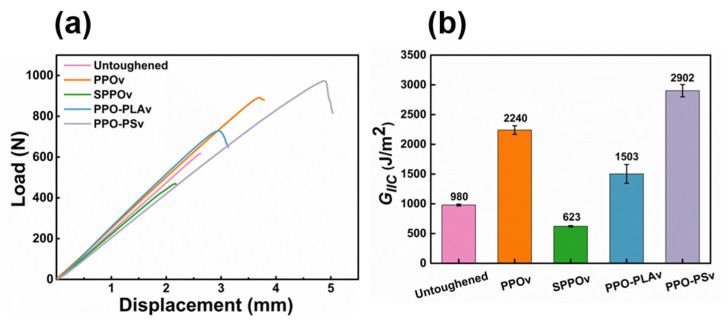
Results of ENF test on toughened laminates of four different electrospun veils: (**a**) load–displacement curves; (**b**) values of *G_IIC_*.

Figure 7 presents the ENF test results. As shown in Figure 7a, the untoughened specimen exhibited a rapid load drop immediately after crack initiation, indicating accelerated crack propagation upon extension. In contrast, the toughened systems (PPOv, PPO-PLAv, and PPO-PSv) demonstrated slower load decay rates and significantly higher critical loads compared to the untoughened system, particularly PPO-PSv, suggesting enhanced crack propagation resistance through interfacial toughening mechanisms. Consistent with the DCB test observations, SPPOv displayed the lowest critical load and loading-point displacement among all specimens.

The *G_IIC_* values are quantified in Figure 7b. The untoughened laminate exhibited a baseline *G_IIC_* of 980 J/m^2^. The PPO-PSv system achieved the highest toughness value of 2902 J/m^2^, representing a 196% increase over that of the untoughened system and a 29.5% improvement relative to that of PPOv (2240 J/m^2^). The PPO-PLAv specimen displayed intermediate fracture resistance, with a *G_IIC_* of 1503 J/m^2^, whereas the SPPOv system showed significantly compromised performance at 623 J/m^2^. This marked reduction in *G_IIC_* confirmed that sulfonated PPO adversely affected the interlaminar interface integrity.

### 3.4. Toughening Mechanism Investigation

The fracture surface morphologies of both DCB and ENF specimens were examined via SEM to elucidate the failure mechanisms and toughening effects of modified/blended PPO electrospun fiber veils under mode I and II loading conditions.

#### 3.4.1. SEM of Mode I Fracture Surfaces

**Figure 8:**
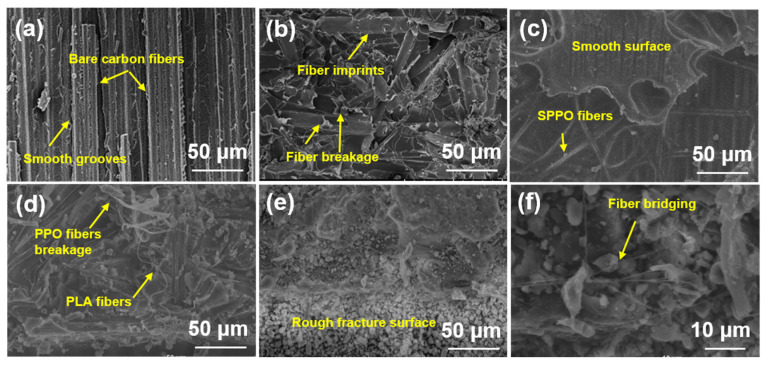
SEM images of fracture surface of DCB samples: (**a**) untoughened sample; (**b**) PPOv sample; (**c**) SPPOv sample; (**d**) PPO-PLAv sample; (**e**,**f**) PPO-PSv sample.

Figure 8 displays representative SEM micrographs of mode I fracture surfaces for untoughened, PPOv, SPPOv, PPO-PLAv, and PPO-PSv specimens. The untoughened specimen (Figure 8a) exhibited a smooth fracture surface with completely separated carbon fibers and brittle epoxy matrix fractures between adjacent fiber bundles. This observation indicates that fiber/matrix debonding and brittle matrix failure constitute the dominant damage modes during mode I delamination of untoughened laminates. The PPOv specimen (Figure 8b) demonstrated a highly roughened fracture surface devoid of exposed carbon fibers, with distinct PPO fiber imprints observable. This morphology suggests that crack propagation primarily occurred within interlaminar regions. Extensive fiber fracture and pull-out were evident, as manifested by residual broken fibers in matrix grooves and blurred groove edges, confirming a strong PPO fiber/matrix interfacial bonding. The predominant toughening mechanisms included fiber bridging and controlled fiber fracture. The SPPOv specimen (Figure 8c) revealed well-defined carbon fibers enveloped by smooth resin matrices without surrounding matrix debris. Intact SPPO fibers adhered to matrix surfaces without fiber pull-out or bridging, indicative of weak interlaminar bonding, which corroborated the low mode I fracture toughness results. As demonstrated by the TGA data, SPPO underwent a three-stage mass loss, with the first stage (50–120 °C) corresponding to moisture evaporation. Notably, the prepreg curing temperature (120 °C) coincided with this temperature range, suggesting potential interference with the resin curing kinetics. The PPO-PLAv specimen (Figure 8d) exhibited phase-separated morphologies, with coarse PPO fibers and fine PLA fibers distributed across the fracture surface. Flattened PPO fibers showed pull-out and fracture signatures, accompanied by irregular fiber imprints and plastic deformation at the fracture tips. In contrast, the PLA fibers remained relaxed without matrix adhesion, indicating negligible energy absorption during crack propagation and further confirming their non-contributory role in toughening. This interfacial incompatibility between amorphous PPO and semi-crystalline PLA, combined with the reduced effective volume fraction of PPO due to PLA blending, explains the lower fracture toughness of PPO-PLAv compared to PPOv.

The PPO-PSv specimen (Figure 8e) displayed the roughest fracture surface morphology, featuring tortuous crack paths that increased the specific surface area and promoted energy dissipation through crack deflection-induced out-of-plane stress redistribution (mechanistically linked to the bending moments at non-planar crack kinks, as reported in [5]) and epoxy matrix plasticity in resin-rich zones. High-magnification imaging (Figure 8f) revealed that the PPO-PS fibers bridged the crack interfaces, with morphological evidence of localized fibrillation confirming energy dissipation through interfacial stress transfer. These fibers suppressed crack propagation by redistributing stress across the laminate thickness, thereby enhancing the interlaminar fracture toughness. The complexity of the crack paths aligns with the heterogeneous toughening framework [33], where energy dissipation is dominated by stress redistribution and fiber bridging mechanisms.

A comparative analysis of mode I fracture toughness rankings and surface roughness (Figure 8e,b,d,a,c corresponding to PPO-PSv, PPOv, PPO-PLAv, untoughened laminate, SPPOv) revealed a direct correlation between fracture surface roughness and toughening performance. Enhanced fracture toughness consistently corresponded to increased surface irregularity, reflecting more efficient energy dissipation mechanisms.

#### 3.4.2. SEM of Mode II Fracture Surfaces

**Figure 9:**
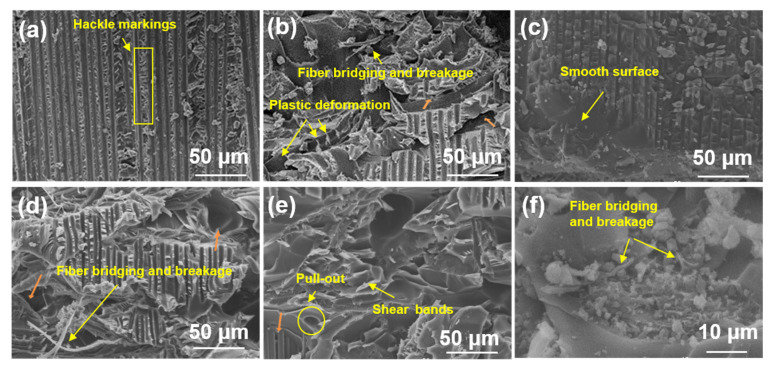
SEM images of fracture surface of ENF samples: (**a**) untoughened sample; (**b**) PPOv; (**c**) SPPOv; (**d**) PPO-PLAv; (**e**,**f**) PPO-PSv.

Figure 9 presents the mode II fracture surface morphologies of representative specimens. The untoughened specimen (Figure 9a) exhibited numerous serrated resin fibrils on the epoxy-dominated fracture surface. These fibrils formed through microcrack nucleation perpendicular to normal stress in resin-rich interlaminar regions, characteristic of shear-dominated mode II fracture [34]. Prior to fiber bridging activation, crack propagation was confined to short distances, with limited energy dissipation. Figure 9b illustrates the fracture morphology of the PPOv specimen, highlighting the serrated, notch-like protrusions resulting from matrix failure. The resin fracture surface appeared rough, exhibiting plastic tearing features, with the interlayer exposed. Crack propagation in the Z-direction was evident at the epoxy–carbon fiber interface and within resin layers containing toughened PPO fibers, as indicated by the orange arrows. During mode II loading, shear forces at the top and bottom surfaces induced plastic flow in the resin matrix, significantly dissipating the fracture energy through extensive viscoelastic deformation [35]. This phenomenon was accompanied by the fracture and plastic deformation of the PPO fibers under shear stress, which further contributed to the material’s enhanced toughness. The SPPOv specimen (Figure 9c) showed smooth fracture surfaces with minimal resin adhesion on carbon fibers and blunted fibril tips, indicating weak interfacial bonding. This facilitated effortless in-plane crack propagation, consistent with its exceptionally low mode II interlaminar fracture toughness. The PPO-PLAv specimen (Figure 9d) revealed exposed interlayers and highly irregular fracture planes, indicating crack propagation at multiple heights [4,36]. As cracks propagated from the resin/fiber interface into the toughened interlayers, flattened PPO fibers exhibited both plastic deformation and bridging. This interaction required additional energy to deform the fibers, causing noticeable crack deflection and significant energy dissipation. The toughening effect, driven by fiber fracture and shear-induced fibril formation, was also present here. Unlike the untoughened specimens, the shear-induced fibrils had a larger effective surface area, enabling enhanced energy absorption through matrix plasticity. Although the fracture morphology of PPO-PLAv was similar to that of PPOv (Figure 9d vs. Figure 9b), the reduced PPO content in PPO-PLAv led to less effective toughening, as evidenced by less severe surface damage. The PPO-PSv sample (Figure 9e,f) exhibited an extensive resin residue, characterized by a scaly shear band morphology with obscured carbon fibers (Figure 9e). This fracture pattern suggests that the high toughness was due to a multiscale energy dissipation mechanism. Firstly, the PPO-PS fibers maintained the interfacial load-bearing capacity, effectively suppressing catastrophic interlaminar debonding. Secondly, the matrix shear yielding (scaly bands) consumed a substantial amount of fracture energy through viscoplastic deformation. Finally, the synergistic interaction between the fibers and the matrix forced the crack to propagate in a serpentine manner within the resin-rich regions, rather than along the weak interface, thus preserving the integrity of the overall composite.

**Figure 10:**
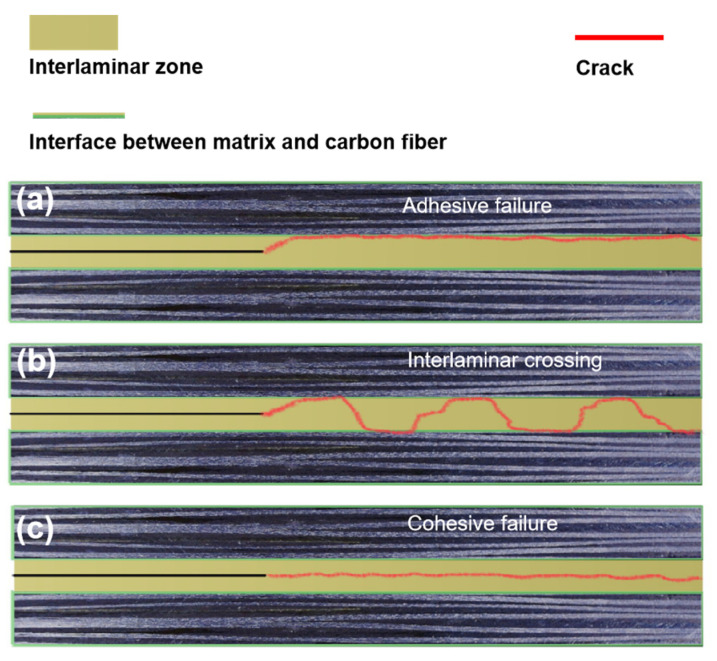
Possible crack propagation paths for different laminates: (**a**) adhesive failure; (**b**) interlaminar crossing; (**c**) cohesive failure.

The ranking of mode II fracture toughness was as follows: PPO-PSv, PPOv, PPO-PLAv, untoughened, and SPPOv. For the untoughened sample, which exhibited poor fracture toughness, crack propagation occurred mainly in plane and along the interface between the matrix and the carbon fibers, without involving Z-phase propagation. This resulted in adhesive failure, as shown in Figure 10a.

In contrast, the samples with higher fracture toughness (PPO-PSv, PPOv, and PPO-PLAv) exhibited rough fracture surfaces and significant interlaminar crossing, as shown in Figure 10b. This suggests that the cracks propagated in a Z-phase manner within the resin-rich layers containing the toughening phase, exhibiting a “saw-tooth” propagation pattern. These “saw-tooth” interlaminar crossings not only increased the total crack propagation length but also triggered various energy dissipation mechanisms along the crack growth path. These mechanisms included pull-out, fracture, and plastic deformation of the veils, bridging of carbon fibers during interlayer crack propagation, debonding between carbon fibers/veils and resin, matrix resin fracture, and carbon fiber pull-out and fracture. These processes greatly enhanced the material’s fracture toughness.

Similarly, the SPPOv sample also demonstrated in-plane crack propagation, but the cracks primarily propagated along the interface between the toughening phase with poor bonding and the matrix. No exposed carbon fibers were visible on the surface, and the failure was cohesive, as depicted in Figure 10c.

## 4. Conclusions

Through sulfonation modification and blending with PLA and PS, which exhibited excellent electrospinning performance, this study successfully explored strategies for improving the electrospinning behavior of PPO and further evaluated its application in the interlayer toughening of composites. The experimental results indicated that both sulfonation and blending of PPO with PLA and PS significantly enhanced the electrospinning performance of PPO, manifested in the reduction of the electrospun fiber diameter. Notably, blending PPO with PLA resulted in a pronounced phase separation, whereas PPO and PS exhibited good blending compatibility, which contributed to the uniformity and structural stability of the fibers. Nevertheless, neither sulfonated SPPO nor the PPO-PLA blended electrospun veils achieved the objective of improving the toughening efficacy of the PPO veils. A quantitative analysis revealed that SPPOv exhibited significantly reduced mode I fracture toughness, with *G_IC_^ini^* and *G_IC_^pre^* values of 151 J/m^2^ and 156 J/m^2^, respectively, compared to 221 J/m^2^ and 207 J/m^2^ for the untoughened laminates. Its mode II interlaminar fracture toughness (*G_IIC_* = 623 J/m^2^) was also inferior to that of the untoughened laminates (980 J/m^2^). This performance degradation may be attributed to a compromised thermal stability of SPPO, which induced defect formation within the laminates. Although PPO-PLAv demonstrated a moderate improvement in *G_IC_^ini^* (376 J/m^2^) and *G_IC_^pre^* (344 J/m^2^) with respect to the untoughened samples, these values represented 13% and 19% reductions compared to those recorded for PPOv (433 J/m^2^ and 426 J/m^2^). *G_IIC_* (1503 J/m^2^) showed a 33% decrease relative to that of PPOv (2240 J/m^2^). Strikingly, the PPO-PS hybrid system manifested remarkable toughening effects. The PPO-PSv system achieved exceptional *G_IC_^ini^* (713 J/m^2^) and *G_IC_^pre^* (688 J/m^2^), representing 223% and 232% enhancements over the values of the untoughened specimens, with further 65% and 61.5% improvements compared to the values obtained for PPOv. *G_IIC_* (2902 J/m^2^) surpassed the value of the untoughened samples by 196% and exceeded that of PPOv by 30%. Notably, the relative interlaminar toughness enhancements significantly exceeded the typical 20–150% improvement range documented for thermoplastic veil systems [37], highlighting the potential advantage of PPO-PS veils in low-toughness matrix applications. The SEM fracture morphology analysis further revealed that the high-toughness samples activated multiple energy dissipation mechanisms, including fiber pull-out, breakage, plastic deformation, carbon fiber bridging, resin debonding, and matrix fracture, during crack propagation. In contrast, the poorly toughened samples predominantly failed via interfacial debonding and adhesive fracture. These findings conclusively highlight the exceptional interlaminar toughening performance of PPO-PS blends, positioning them as promising candidates for high-performance composites.

## Figures and Tables

**Table 1:** Electrospinning parameters used to obtain different veils.

Types of Veils	Polymer/Solvent (% *w*/*w*)	Voltage (kV)	Tip to Collector Distance (cm)	Flow Rate (mL/h)
PPO	18% PPO + 82% CHCl_3_	18	18	0.5
SPPO	12% SPPO + 88% CHCl_3_	16	25	1
PPO-PLA	6% PPO + 6% PLA + 88% CHCl_3_	16	20	1
PPO-PS	8.5% PPO + 8.5% PS + 83% CHCl_3_	16	20	1

## Data Availability

Data are contained within the article.
